# Occurrence of *Vibrio* spp. in Selected Recreational Water Bodies in Belgium during 2021 Bathing Season

**DOI:** 10.3390/ijerph20206932

**Published:** 2023-10-17

**Authors:** Rosalie Sacheli, Camille Philippe, Cécile Meex, Samy Mzougui, Pierrette Melin, Marie-Pierre Hayette

**Affiliations:** Department of Clinical Microbiology, Belgian National Reference Center *Vibrio cholerae* and *Vibrio parahaemolyticus*, Center for Interdisciplinary Research on Medicines (CIRM), University Hospital of Liege, 4000 Liège, Belgium; camille.philippe@sciensano.be (C.P.); c.meex@chuliege.be (C.M.); samy.mzougui@chuliege.be (S.M.); pierrette.melin@uliege.be (P.M.); mphayette@chuliege.be (M.-P.H.)

**Keywords:** *Vibrio* spp., vibriosis, Belgium, recreational water, bathing water

## Abstract

In recent years, a global increase in the number of reports of human vibriosis involving *V. cholerae* non-O1/O139 (NOVC) and other *Vibrio* spp. has been observed. In this context, the Belgian National Reference Center for *Vibrio* conducted an assessment of the presence of *Vibrio* spp. in recreational waters. Water sampling was performed monthly in different lakes in Wallonia and Flanders, including the North Sea. The collected water was then filtrated and cultured, and *Vibrio* spp. was quantified according to the Most Probable Number (MPN). Presumptive colonies were confirmed via MALDI-TOF, and PCR for virulence genes was applied if justified. No *Vibrio* spp. was found in the analyzed water bodies in Wallonia. However, NOVC was isolated from three different lakes in Flanders and from coastal water. In addition, *V. alginolyticus* and *V. parahaemolyticus* were also detected in coastal water. No clear impact of the pH and temperature was observed on *Vibrio* spp. occurrence. Our study demonstrates the presence of *Vibrio* spp. in different bathing water bodies, mostly in the north of Belgium, and supports the recommendation to include *Vibrio* spp. as a water quality indicator for bathing water quality assessment to ensure the safety of water recreational users in Belgium.

## 1. Introduction

Infections caused by pathogenic *Vibrio* spp. are a serious health problem. Most of these infections are due to the consumption of undercooked seafood or following contact with contaminated waters. Regarding *Vibrio cholerae* (*V. cholerae*), infections are classified into cholera and non-cholera regarding whether the isolated strain belongs to type O1 or O139 and is producing the cholera toxin or not. Although inducing cholera, *Vibrio cholerae* non-O1 non-O139 (NOVC) and other *Vibrio* spp. may also cause severe human diseases. Vibriosis is defined as illness caused by *Vibrio* species other than *V. cholerae* O1 and O139. People who are more vulnerable to severe vibriosis are immunocompromised people or those with underlying diseases, such as liver disease, cancer, diabetes, or people who have recently undergone stomach surgery. Non-toxigenic *V. cholerae* and most *Vibrio* spp. are found in aquatic environments and are generally non-pathogenic. *Vibrios* are able to grow over a wide temperature range (from 20 °C to >40 °C), tolerate a range of salinities, and tend to grow best under alkaline conditions, although most species of *Vibrio* will grow between pH 6.5 and 9.0 [1]. NOVC has been associated with extra-intestinal infections, including bacteremia and sepsis, and skin and soft tissue infections, such as wound infections, cellulitis and necrotizing fasciitis, peritonitis, cholecystitis, endophthalmitis, ear infections, urinary tract infection, and meningitis [2,3,4,5,6,7,8,9]. The vibrioses identified in Belgium have been associated with recreational bathing in lakes and marine water [10]. Recently, reports of human infections, which can be life-threatening, involving NOVC and other *Vibrio* spp. have been identified in France and also from other European regions, such as in northern Germany and the Baltic Sea coastline [2,6,11,12,13]. Non-cholerae vibriosis cause an estimated 80,000 illnesses and 100 deaths in the United States of America every year [14]. A recent report from Korea describes a case of vibriosis in a 52-year-old man without any underlying disease who was diagnosed with NOVC infection and died within 72 h after admission to an intensive care unit [15]. Regarding other *Vibrio* spp. such as *V. parahaemolyticus*, *V. damsela*, *V. fluvialis*, and *V. algynolyticus*; they can cause sporadically human illnesses originating in water, such as wound infections, otitis, gastroenteritis, bacteremia, and sepsis [16,17,18,19,20,21,22,23]. Rarely, they cause food poisoning outbreaks. The CDC noted the fact that some *Vibrio* species, such as *V. vulnificus*, can induce severe and life-threatening infections. *V. vulnificus* infections often require admission to an intensive care unit or lead to limb amputations. About one in five people with this invasive infection die, sometimes within 48 h [14,24]. In Belgium, as recommended in the European Union, water for recreational/bathing use, such as lakes and coastal water, are not yet monitored for *Vibrio* spp. but are well monitored for fecal indicators if bathing is authorized. Among the main fecal indicators of interest, *Escherichia coli* and intestinal *Enterococci* are determined. In Wallonia, the “Institut scientifique des services publics” (ISSeP) oversees water quality surveillance; in Flanders, the quality of the surface water is measured and controlled by the Flanders Environment Agency (abbreviated VMM). We assume that other bacteria, such as *Vibrio* spp., should also be monitored in waters as they can rapidly become a public health issue. Little is known about the abundance, pathogenicity, and ecology of *Vibrio* spp. in European waters. At the Belgian National Reference Center for *V. cholerae* and *parahaemolyticus* (NRC), we decided to conduct a pilot study by performing the cartography of Belgian water bodies to determine the presence of *Vibrio* spp. A few selected areas were screened to evaluate the risk of human contamination by *Vibrio* spp. through recreational/bathing Belgian waters. Global warming has been linked to *Vibrio* occurrence in some studies [12,13]. The ECDC (the European Centre for Disease Prevention and Control) developed the tool “*Vibrio* map viewer” that can serve as an early warning system, as it is thought that the number of *Vibrio* spp. infections will increase in the next years due to climate change [25]. Taking into account that global warming leads to a higher probability of *Vibrio*-associated infections in Belgium, this pilot study is, despite its limitations, of high scientific relevance to public health.

## 2. Material and Methods

### 2.1. Design of the Study

**Figure 1:**
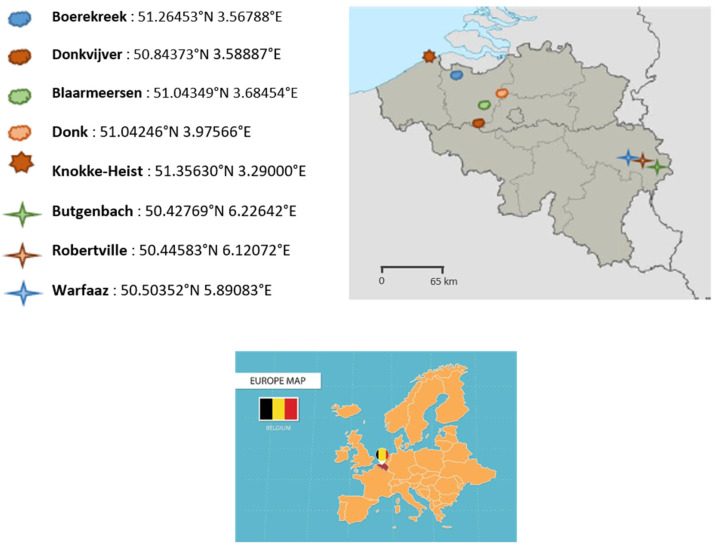
The geographical location and postal code of the eight Belgian bodies of water screened for the presence of *Vibrio* spp. in 2021 and the location of Belgium inside Europe. Source: http://www.carte-du-monde.net (accessed on 13 September 2023). (Belgium map) and Vecteezy (Europe map).

According to recent Belgian clinical cases of vibriosis and to the distribution of recreational/bathing water locations, 8 sites were selected in Wallonia and Flanders, including the North Sea (marine water). Figure 1 illustrates the different locations in Belgium selected for water analysis.

### 2.2. Sampling

From May to September 2021, one water sample was collected monthly at each of the eight sites, resulting in a total of 40 water samples. According to the official bathing period and EU directives recommendations, water collection was performed from May to September 2021 [26]. A telescopic device (VWR, Radnor, PA, USA) was used for the collection of 1 L water samples. Water samples were collected in sterile containers (VWR, Radnor, PA, USA) and then kept on melting ice in an isotherm box during transportation to the NRC laboratory and kept at 4 °C upon arrival.

All the samples were analyzed within 24 h. The pH and temperature of each water sample were measured in situ using a 7011 microprocessor-based waterproof pH/mV/temperature tester (Esico International, Parwanoo, India) and recorded on-site during sampling.

### 2.3. Culture and Identification Methods

Our procedure was adapted from the one described by Schets et al. [27]. All the samples were cultured for the detection and quantification of *Vibrio* spp. using the Most Probable Number (MPN) culture method. Specifically, a three-tube–five-dilution MPN format was used; it consists of triplicate serial dilutions (five) in alkaline peptone water (APW, Merck, Darmstadt, Germany) as an enrichment prior to subculturing on thiosulfate citrate bile saccharose agar medium (TCBS, Biorad, Hercules, CA, USA). The cultures were performed as follows: 10 mL and 100 mL were membrane-filtered (0.45 µm pore, Merck, Darmstadt, Germany), and the filters were then inoculated in 50 mL of APW. In parallel, three non-filtrated water samples of 10 µL, 100 µL, and 1 mL of collected water were directly inoculated in 9 mL tubes of APW (Lustiner, France). All the inoculated APW samples were incubated at 41 °C for 18–20 h, and after incubation, 10 µL of each APW sample was sub-cultured on TCBS (Biorad, Hercules, CA, USA) and incubated at 35 °C for 16–20 h. The positive TCBS plates obtained from the serial dilutions in APW were used for the estimation of the concentration of *Vibrio* spp. in the different samples according to the MPN method and guidelines [28,29].

The identification of colonies that grew on TCBS medium was conducted using MALDI-TOF mass spectrometry (MALDI Biotyper, Bruker Daltonics, Billerica, MA, USA) according to the manufacturer’s instructions. For identification, generated spectra were analyzed using the MALDI Biotyper 2.0 software package (Bruker Daltonics, Billerica, MA, USA) and compared to the reference spectra included in both regular and bioterrorism libraries. Five colonies per positive TCBS plate were tested. If *V. cholerae* was identified, agglutination tests were performed for serogroup/serovar, O1, and O139 determination following the manufacturer’s instructions (Mast assure, Toulon, France).

### 2.4. Molecular Detection of Toxin Genes in V. cholerae and V. parahaemolyticus

For each isolate identified as *V. cholerae*, molecular detection using real-time PCR for the *V. cholerae CtxA* gene was performed according to the PCR protocol described by Blackstones et al. [30]. Briefly, genomic DNA was extracted using the Maxwell 16 SEV cell kit (Promega, Madison, WI, USA). Approximately 1 ng of DNA was used in the PCRs using primers and conditions as previously described [30]. Real-time PCR was performed using an LC 480 thermocycler (Roche, Basel, Switzerland). When a *Vibrio parahaemolyticus* strain was identified, thermostable direct hemolysin (*tdh*) and thermostable-related hemolysin (*trh*) were detected using classical PCR as described by Kaysner et al. 1999 [31]. A Veriti thermal cycler (ThermoFisher Scientific, Waltham, MA, USA) was used, and the presence of amplicons was revealed via agarose gel electrophoresis (Thermofisher Scientific, Waltham, MA, USA).

### 2.5. Salinity

Salinity was not directly measured during this study. However, conductivity values were provided by the “Vlaamse Milieumaatschappij Sturing en Rapportering Water Monitoring waterkwaliteit” and from the “Service public de Wallonie” department of environment and water. The available results of salinity/conductivity are reported in Table S2. Values for Flanders lakes are available for our studied period (May–September 2021) for Donkvijver, Blaarmeersen, and Boerekreek. For Donk, only the salinity/conductivity values from the year 2022 were available and are described in Table S2. For the North Sea (Knokke Heist), the measure of salinity was not conducted by the “Vlaamse Milieumaatschappij”, but we know that the salinity of the North Sea is quite stable and is around 35 PSU (1 PSU = 1 g NaCl/kg of water) [32].

### 2.6. Statistics

To study the abundance of *Vibrio* according to its importance (3 groups, no growth, ≤110 CFU/mL, >110 CFU/mL), an ordinal logistic regression model was used. A Student *t*-test was used to compare the pH and temperature values between Flanders and Wallonia. An asymptotic Spearman correlation test was performed to correlate the salinity with the *Vibrio* concentration. The results are considered significant at the 5% level of uncertainty (*p* < 0.05). Calculations were performed using SAS version 9.4. (SAS, Cary, NC, USA)

## 3. Results

### 3.1. Vibrio Detection and Quantification

*Vibrio* spp. was detected in the water samples from the Belgian coastal water and from three lakes out of four tested inland waters in Flanders. *Vibrio* spp. was not found in the Wallonian lakes, and Donk Lake analyzed in Flanders. Regarding the identification of the positive cultures, *V. cholerae* (non-01 and non-0139 determined using the agglutination test) was identified in the three studied lakes in Flanders and in the coastal water. Other *Vibrio* spp., *V. alginolyticus,* and *V. parahaemolyticus* were also found in the coastal water. All *V. cholerae* strains were characterized for the presence of the cholera toxin (the targeted gene was CtxA) using real-time PCR, but all were negative. The PCR targeting of hemolysins *tdh*/*trh* was applied to all the *V. parahaemolyticus* strains, but the results were also negative. No further tests were conducted for the *V*. *alginolyticus* strains. Table 1 summarizes all the positive and negative cultures in the different water bodies. Identification of positive cultures from different water bodies was achieved via the MALDI-TOF.

**Figure 2:**
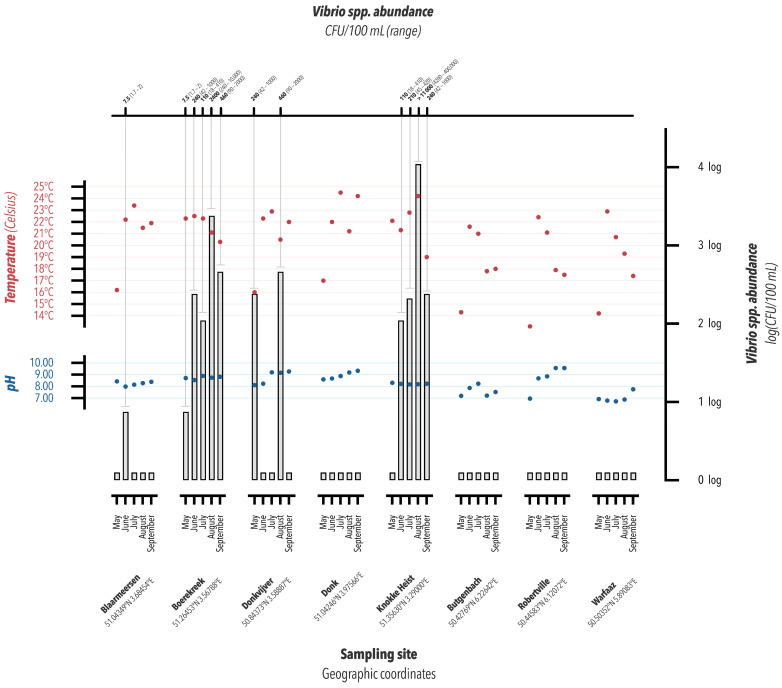
Graphical representation of the estimation of the concentration in CFU/100 mL of *Vibrio* spp. (with ranges in brackets, which are given in the log scale on the right) determined with the MPN method in different water bodies merged with the distribution of the pH values (in blue) and temperature records (in red) measured in each water body from May to September 2021.

The calculated concentrations obtained using the MPN method are described in Figure 2. For each considered water body/period, the results of fecal bacterial contamination are available for some water bodies; follow these links: http://www.kwaliteitzwemwater.be (accessed on 7 September 2023) and http://environnement.wallonie.be/baignade (accessed on 7 September 2023). The available results are described in Table S2. All the values were under the threshold values for a very good or acceptable quality of water. These are ≤400 CFU/100 mL for enterococci and ≤1000 CFU/100 mL for *E. coli* for very good quality of recreative freshwater and ≤700 CFU/100 mL for enterococci and ≤2000 CFU/100 mL for *E. coli* for an acceptable quality. Regarding these last thresholds, swimming is still allowed but not recommended for young children and vulnerable people. For marine water, the values are ≤400 CFU/100 mL for enterococci and ≤1000 CFU/100 mL for *E. coli* (https://kwaliteitzwemwater.be/nl/normen, accessed on 7 September 2023 No data were available for Warfaaz Lake and Donk Lake for the studied period.

### 3.2. pH and Temperature Monitoring

The pH of each water sample was measured in each location at the time of sampling. The results are represented in Figure 2. The average pH value was 7.8 and 8.6 in Wallonia and Flanders, respectively. A Student *t*-test showed a statistically significant difference in pH in Flanders than in Wallonia (*p* = 0.0009). There was no statistically significant impact due to the pH variation on NOVC occurrence according to logistic regression (*p* = 0.19). In Donk Lake, although the pH value was, on average, 8.79, no *Vibrio* spp. was found, while in the North Sea, where the average pH value was lower (8.22), high levels of *Vibrio* spp. were observed (see Figure 2). However, we noticed that the average pH was lower in Wallonia than it was in Flanders, and no *Vibrio* spp. was found in Wallonia.

Water temperatures were also recorded, and the results are illustrated in Figure 2. The average water temperature in Wallonia was 18.6 °C, while it was 21.4 °C in Flanders. Student *t*-test indicated a statistically significant difference between the temperature of the water in Flanders and that in Wallonia (*p* = 0.0019), which was linked to the months of the year (it was lower in May than it was in summer months, *p* = 0.0001). Ordinal logistic regression showed that the temperature had no statistically significant impact on *Vibrio* spp. occurrence (*p* = 0.19). Table S1 summarizes all the temperature, pH, and *Vibrio* spp. MPN data in the different water bodies.

### 3.3. Salinity

Regarding the available values, the mean value of salinity for Boerekreek (1.4) was higher than it was in the other freshwaters (0.21 PSU for Blaarmersen, 0.23 PSU for Donkvijver, and 0.20 PSU for Donk).

For Walloon lakes, measurements of salinity were available only for the year 2019 for Butgenbach and Roberville. The salinity value was calculated to be 0.05 and 0.06 PSU, respectively. No salinity measurement was available for Warfaaz Lake. The asymptotic Spearman correlation test showed that salinity and *Vibrio* concentrations were positively correlated (*p* = 0.00001, correlation coefficient = 0.69).

## 4. Discussion

In our study, we monitored *Vibrio* spp. in a selection of key inland and coastal Belgian water bodies. This has not been achieved for a long time, as previous observations in Belgium were performed in 1985 [33]. It is well known that the presence and growth of *Vibrio* spp. in water depends on multiple environmental factors. Although the effects of these parameters are highly species-dependent, in general, temperature and salinity are considered to be linked with *Vibrio* growth [34,35,36]. The other parameters implicated in *Vibrio* growth include nutrients, chlorophyll-a concentration, and pH [37,38].

Our study shows that warm temperatures favor *Vibrio* spp. growth, as more bacteria were found in waters with an increased temperature, even if this was not statistically significant. The mean temperature in Walloon waters was lower than that in Flanders, which can maybe partially explain the fact that no *Vibrio* spp. was found in Walloon waters. The link between *Vibrio* spp. growth and temperature increase have already been demonstrated in previous studies [39,40]. It has been described that *Vibrio* spp. can survive in water (viable but not culturable) during the winter period when the environmental conditions are not favorable, allowing it to survive in the environment. *Vibrios* can attach to copepods to persist in the environment. They can then be released in the late months of spring when the temperatures are higher than 15 °C and the environmental conditions are more favorable for *Vibrio* development [41,42,43].

In our study, the pH value does not seem to impact bacterial growth. It is well known that *Vibrio* spp. grow better in an alkaline environment, although most species of *Vibrio* will grow between pH 6.5 and 9.0 [1]. High pH values were measured in Donk Lake without any bacterial growth. However, the pH of lakes in Wallonia is lower than that in Flanders, which could partially explain why no *Vibrio* spp. was found in Walloon lakes. Another parameter may have influenced the fact that no *Vibrio* spp. was found in Wallonia as, for example, it is far away from the North Sea. Indeed, several studies provide evidence that aquatic birds may act as carriers and disseminate *V. cholerae* and other *Vibrio* spp. over a wide area [44,45]. As Walloon lakes are further away from the North Sea, this can probably be one cause of the absence of *Vibrio* spp. in this area rather than in Flanders lakes, which are closer to the North Sea. However, this hypothesis does not explain why no *Vibrio* spp. was found in the lake, which is near to the other lakes in Flanders and the North Sea.

Salinity was not directly measured in our study; this is a major limitation of this study. However, some data were available from the “Vlaamse Milieumaatschappij Sturing en Rapportering Water Monitoring waterkwaliteit” and the “Service public de Wallonie” department of environment and water. We saw that, in Boerekreek, where *Vibrio* spp. was isolated during sampling (from May to September), the salinity was a little bit higher (1.4) than it was in the other freshwater bodies (around 0.2), which can maybe explain the fact that we found more *Vibrio* in this site. However, *Vibrio* spp. has also been found sporadically in Donkvijver and Blaarmeersen with very low salinity values (0.21 and 0.23, respectively). The salinity in Wallonia was around 0.055 (data not known for Warfaaz Lake), which is also very low and can explain the total absence of *Vibrio* spp. in these water bodies. Some other parameters, such as the natural environment of the lake and the presence of algae as a nutrient and chlorophyll-*a* concentrations, may also influence *Vibrio* growth [37,38]. An in-depth analysis of the water is necessary to better understand what inhibits *Vibrio* spp. growth in some environments.

The low number of analyzed water bodies is also a limitation of this study. We consider that this was a pilot study conducted to learn if there was a need to check water for *Vibrio* spp. presence at the national level. Despite the few water bodies tested, the major information to retain is that we found *Vibrio* spp. in these tested lakes, meaning that, certainly, other water bodies in Belgium will also contain *Vibrio* spp. and that the monitoring of bathing water for these bacteria is necessary.

Currently, the number of reported cases linked to recreational water-related *Vibrio* illnesses in Belgium is low, but infections do occur. *Vibrio* spp. infections have a clearly marked seasonal distribution; they are mostly reported during summer and early autumn, corresponding to periods with warmer temperatures [46]. In the summer of 2018, one Belgian strain of NOVC was isolated from an 8-year-old girl hospitalized with acute gastroenteritis. The patient was swimming and accidentally drank water in a recreational area located in Flanders, Belgium. Large-volume water samples were then analyzed from this place, and NOVC was isolated from this aquatic environment, ranging from 4 to 100 CFU/100 mL (data from the Belgian NRC, not published). Another Belgian case has been described and published concerning a 45-year-old man without any medical history who developed bacteremia with NOVC in 2017. This patient’s history revealed that the patient swallowed a huge amount of water during a fall while paddling in a brackish Belgian recreational creek. The water samples taken from the creek in June 2017 (14 days after exposure) also yielded 5.10^4^–10^5^ CFU/100 mL of NOVC [10]. These cases highlight the importance of monitoring *Vibrio* spp. in Belgian waters and illustrate the fact that the presence of *Vibrio* spp. can be linked to human infections.

In our study, *Vibrio* spp. was quantified using the MPN method, and concentrations above 1.1 × 10^4^ CFU/100 mL were estimated. *V. cholerae* require 1 × 10^3^ to 1 × 10^8^ cells in the inoculum to successfully give a host gastroenteritis [47]. For immunocompromised persons and for those with other infections caused by *Vibrio* spp., no dose–response data are available. The concentration of *Vibrio* spp. found in Belgian waters was revealed to be sufficient enough to infect humans and cause gastroenteritis or other infections.

Different studies have already shown the presence of *Vibrio* spp. in European waters [35,36,40,48]. More precisely, a German team monitored *V. cholerae*, *V. parahaemolyticus*, and *V. vulnificus* at seven recreational bathing areas from 2017 to 2018, including during a heat wave event in the summer of 2018. They observed that all three *Vibrio* species were present in the water and sediment samples at all the sampling sites and that temperature was the main driving factor of *Vibrio* occurrence, whereas *Vibrio* community composition was mainly modulated by salinity. Due to this factor, they observed that the dominant *Vibrio* species found in the North Sea was *V. parahaemolyticus*, whereas *V. vulnificus* was mostly found in the Baltic Sea samples [13]. As mentioned, salinity was not measured in our study, but regarding values given by the “Vlaamse Milieumaatschappij Sturing en Rapportering Water Monitoring waterkwaliteit” by the “Service public de Wallonie” department of environment and water and data found in the literature, the salinity level is higher in the North Sea than it is in freshwaters. Indeed, the salinity of the North Sea has been estimated to be around 35 PSU, while the salinity of freshwater is, in general, lower than 1.5 PSU. [32,49]. Interestingly, while *V. cholerae* is known to prefer a salinity around 15 PSU, it was frequently found in the North Sea with a higher salinity level and also in freshwater with a lower salinity level. *V. parahaemolyticus* and *V. alginolyticus* have also been isolated from the North Sea but not from freshwaters, which is not surprising as they are well known to prefer halophilic environments. One Spanish study indeed revealed the important role of salinity in driving the seasonal pattern and the spatial distribution of *V. parahaemolyticus* in the marine environment of the Atlantic coast in Europe [36]. Our results are in accordance with observations in a French study in 1999, where *V. alginolyticus*, *parahaemolyticus*, and NOVC were found in coastal waters. However, they also found *V. vulnificus*, which was not isolated in our study [50]. A study conducted in the UK used sea surface temperature data around English and Welsh coastlines to identify places with favorable conditions for *Vibrio* spp. growth. The shellfish samples collected from three locations showed that the presence of *Vibrio* spp. was positively associated with an increase in sea surface temperature. Among the isolated *Vibrio* spp., *Vibrio parahaemolyticus* was isolated, but two other species that had never been isolated before in UK waters were found, namely, *V. rotiferianus* and *V. jasicida,* which are important aquaculture pathogens [51]. This example shows that global warming and warming sea surface temperatures have led to an increase in the prevalence of *Vibrio* spp. in waters, especially in temperate regions, and our work shows that this is also true for inland waters. In 2009, four bathing sites (inland waters) in The Netherlands were monitored for potentially human pathogenic *Vibrio* species. In this study, the analyzed water bodies were, in general, positive for *Vibrio* from May to October. Although they observed that more samples were positive for *Vibrio* at elevated water temperatures, a quantitative relation between *Vibrio* numbers in water samples and the water temperature was not observed, as in our study [27]. In a paper written by Sterk et al., data on the occurrence of *Vibrio* spp. at six different bathing sites (again inland water bodies) in the Netherlands (2009–2012) were described. The conclusion of this study (using an empirical formula) was that increasing the water temperature will probably increase the risk of *Vibrio*-related illnesses in the future due to global warming [52]. In a recent paper from Serbia, the authors investigated the occurrence of NOVC in nine Serbian natural and artificial lakes and ponds. With the exception of one highly saline lake, all the investigated water bodies harbored NOVC with concentrations ranging from 5.4 × 10^1^ to 1.86 × 10^4^ CFU/100 mL. So, these described concentrations are in the same order as what we described in our tested inland waters [53].

Vibriosis linked to water contact is also increasingly reported in Europe [12,54,55]. This concerns NOVC but also other *Vibrio* species. These cases are not to be neglected. Indeed, *V. cholerae* and *V. parahaemolyticus* infections have been described to be fatal in some situations despite the absence of classical virulence factors in these bacteria [15,56,57,58]. A lot of serious cases have also been described to be related to non-cholera *Vibrio,* such as fulminant cellulitis caused by NOVC in Finland [8]. Three hundred and fifty cases of bacteremia caused by NOVC were identified until 2015 in the literature, as reviewed by Deshayes et al. 2015 [2]. Another team reviewed 23 cases of NOVC bacteremia from 2015 to 2019 [59]. They showed that these *Vibrio* spp. present in waters outside cholera-endemic areas pose a potential public health problem, especially to immunocompromised populations. Immunosuppressed patients or those with other predisposing factors such as cirrhosis should be aware of the risk of eating undercooked seafood or swimming in some water areas, especially if they have pre-existing wounds. Clinicians should better inform and educate these patients about this underestimated risk. Serious cases of immunocompetent subjects have also been described [2,10,60], meaning that *Vibrio* spp. surveillance is not just about the immunocompromised population.

The *V. cholerae* strains that we found in Belgian water were all NOVC and non-toxin producers. This was the same for *V. parahaemolyticus*, which does not harbor some virulence factors such as hemolysins. It is frequently reported that environmental strains outside cholera-endemic areas are often not virulent, even if they can cause severe illnesses [61,62,63]. Similarly, while 90% of clinical *V. parahaemolyticus* strains classically harbor *tdh*/*trh* genes, these genes are less common in environmental strains even if some cases have already been described, with “pandemic” strains being positive for *tdh*/*trh* in Northern Adriatic water in Italy [36,48,64]. In Slovakia, one study focusing on aquatic *Vibrio* isolated from freshwaters showed that neither the cholera-toxin-coding gene *ctx*A nor the genes *zot* (zonula occludens toxin), *ace* (accessory cholera toxin), and *tcp*A (toxin-coregulated pilus) were present in any of the 31 studied samples [53].

In Northern Europe, the increase in reported vibriosis cases corresponds to major heatwaves occurring during summer [65]. Concurrently, species like NOVC, *V. vulnificus*, and *V. parahaemolyticus* are increasingly prevalent in Northern waters and seem to follow regional climatic variations, with outbreaks typically occurring after warm temperature periods [65,66,67,68]. Similarly, the samples collected in the last 60 years by the continuous plankton recorder survey showed that the genus *Vibrio*, including the human pathogen *V. cholerae*, has increased in prevalence in the last 44 years along the coast of the North Sea, and this increase is strongly correlated with water warming [69,70]. Climate change is expected to both directly and indirectly affect environmental conditions. Higher atmospheric temperatures will induce higher water temperatures, both in oceans and inland waters. If such extreme conditions occur frequently during future summer, increased numbers of water-related illnesses after bathing should be expected; these water-related illnesses could also give rise to outbreaks. The emergence of *Vibrio* spp. from southern regions to northern waters has been already mentioned [65,69,71,72]. Considering these conditions, a higher incidence of *Vibrio* spp. infections are clearly predicted for the future, and some authors even consider *Vibrio* spp. as the “barometer” of climate change [66].

We assume that the lack of *Vibrio* spp. monitoring in Belgian water is problematic and will be an increasing concern in the future regarding global warming. Our study shows that NOVC and other species of *Vibrio,* such as *V. parahaemolyticus* and *V. alginolyticus,* are present in Belgian waters, which are of clinical interest due to their potential pathogenic impact on humans. These observations reinforce the need for *Vibrio* spp. monitoring in Belgian and, more extensively, in European waters. The EU Directive 2006/7/EC for bathing water monitoring requires control of the presence and density of *Enterococci* and *Escherichia coli* as bacterial fecal contamination indicators. There are no recommendations for the monitoring of *Vibrio* spp. [26]. Our study indicates that fecal indicator bacteria (FIB) are potentially inappropriate indicators for *Vibrio* spp., given that the FIB level did not exceed the regulatory standard for acceptable recreational water quality in the sample water bodies where *Vibrio* spp. were found, reinforcing the need for the specific monitoring for these pathogens in recreational areas. The costs of this monitoring are limited, as only a TCBS medium, alkaline peptone water, and a system of filtration for water with 0.45µm filters are needed for analysis. If PCR is needed for characterizing the CtxA virulence factors, the collected strains can be sent to the Belgian National Reference Center for analysis. These controls for *Vibrio* spp. should be provided, together with the FIB testing by competent organisms of water such as the Vlaamse Milieumaatschappij Sturing en Rapportering Water Monitoring waterkwaliteit” in Flanders and the “Service public de Wallonie” department of environment and water in Wallonia. So, the devices needed, such as the filtration pump, should be regular equipment in control laboratories. There is a crucial need for a greater understanding of the non-cholera *Vibrio* spp. associated risk within a European context. The WHO guidelines already consider *Vibrio* spp. (*V. alginolyticus*, *V. vulnificus*, *V. parahaemolyticus*, and non-O1/O139 *V. cholerae*) as microorganisms of possible concern in recreational waters. Even if these organisms are not present in the current recommendations, the WHO has already alerted the international community about the possible impact of *Vibrio* spp. in recreational waters [73]. The warming of inland waters linked to global warming will probably induce larger numbers of *Vibrio* population and, consequently, an increased risk of *Vibrio*-related infections. The European Centre for Disease Prevention and Control (ECDC) has developed the “*Vibrio* Map Viewer” as a point of attention for the public to help decrease humans’ exposure to *Vibrio*-contaminated coastal waters. This tool monitors the sea surface temperature and salinity in the Baltic Sea during summer to provide alerts about elevated environmental conditions that can lead to a higher risk of *Vibrio* growth and, so, a higher risk of *Vibrio* infections in the population [25]. In our opinion, this kind of surveillance should also be performed in the North Sea, as the Baltic Sea and the North Sea are the fastest-warming seas in Europe [74]. It is important to mention a lack of detailed surveillance information regarding non-cholera *Vibrio* infections in Europe [65]. Indeed, these pathogens are not notifiable in many European countries, which probably softens the real clinical impact of human vibriosis. We now have to consider that future pandemics due to *Vibrio* spp. via water is probable even in Europe, and we have to change our current beliefs and behaviors regarding this class of bacteria.

## 5. Conclusions

Our study demonstrates the presence of NOVC and other *Vibrio* spp. at concentrations that are able to cause human infections in different water bodies in the north of Belgium. The mean temperatures and pH were higher in the selected Flemish water bodies than they were in the selected Walloon lakes. A high pH and a high temperature can be favorable factors for the growth of *Vibrio* spp. Some other factors, such as salinity, should also be included in future surveillance. This study supports the recommendation to include *Vibrio* spp. in water quality controls in aquatic natural environments in order to define if water recreational activities may be conducted with an acceptable risk to humans in Belgium. In light of global warming, we think that monitoring *Vibrio* spp. in waters in Belgium should be mandatory.

## Figures and Tables

**Table 1:** *Vibrio* spp. detection out of samples collected monthly in 8 water bodies in Wallonia and Flanders. Results of TCBS and MALDI-TOF MS identification of cultures (five colonies tested using positive TCBS plate tests).

Region and Type of Body Waters	Site	Month of Collection					Number of Positive Samples Out of 5
		May	June	July	August	September	
Wallonia lakes	Butgenbach	VND	VND	VND	VND	VND	0
	Robertville	VND	VND	VND	VND	VND	0
	Warfaaz	VND	VND	VND	VND	VND	0
Flanders lakes	Donk	VND	VND	VND	VND	VND	0
	Blaarmeersen	VND	VND	*V. cholerae*	VND	VND	1
	Donkvijver	*V. cholerae*	VND	VND	*V. cholerae*	VND	2
	Boerekreek	*V. cholerae*	*V. cholerae*	*V. cholerae*	*V. cholerae*	*V. cholerae*	5
Flanders North Sea (salted water)	Knokke Heist	VND	*V. cholerae* *V. alginolyticus*	*V. cholerae* *V. alginolyticus* *V. parahaemolyticus*	*V. cholerae* *V. alginolyticus* *V. parahaemolyticus*	*V. cholerae* *V. alginolyticus*	4 4 2

VND = Vibrio not detected.

## Data Availability

Not applicable.

## Supplementary Materials

The following supporting information can be downloaded at: https://www.mdpi.com/article/10.3390/ijerph20206932/s1, Table S1: Summary of the measures of Temperature and pH and the estimated enumeration of *Vibrio* spp. by waterpoint and month; Table S2: Values of conductivity in mS/cm and of the fecal indicators *E. coli* and Enterococci given by the “Vlaamse Milieumaatschappij Sturing en Rapportering Water Monitoring waterkwaliteit” and the “Service public de Wallonie, department of environment and water”.
