# Supplementary material for: Occurrence of Vibrio spp. in Selected Recreational Water Bodies in Belgium during 2021 Bathing Season
Source: Int J Environ Res Public Health. 2023 Oct 17;20(20):6932. doi: 10.3390/ijerph20206932 (PMC10606296; doi:10.3390/ijerph20206932)
Supplement: Supplementary file 1 [file ijerph-20-06932-s001.zip › ijerph-2592194-supplementary.pdf]

Table S1: Summary of the measures of Temperature and pH and the estimated enumeration of *Vibrio* spp. by waterpoint and month.

N° Sampling	Region	Location	Month	Temperature (°C)	pH	*Vibrio* spp. CFU/100mL (range)	
1	Flanders	Blaarmeersen	May	16.2	8.43	0	
2	Flanders	Blaarmeersen	June	22.2	7.99	7.5	(1.7 – 2)
3	Flanders	Blaarmeersen	July	23.4	8.15	0	
4	Flanders	Blaarmeersen	August	21.5	8.28	0	
5	Flanders	Blaarmeersen	September	21.9	8.39	0	
6	Flanders	Boerecreek	May	22.3	8.71	7.5	(1.7 – 2)
7	Flanders	Boerecreek	June	22.5	8.54	240	(42 – 1000)
8	Flanders	Boerecreek	July	22.3	8.89	110	(18 – 410)
9	Flanders	Boerecreek	August	21.1	8.74	2400	(420 – 10000)
10	Flanders	Boerecreek	September	20.3	8.82	460	(90 – 2000)
11	Flanders	Donkvijver	May	16	8.11	240	(42 – 1000)
12	Flanders	Donkvijver	June	22.3	8.23	0	
13	Flanders	Donkvijver	July	22.9	9.2	0	
14	Flanders	Donkvijver	August	20.5	9.16	460	(90 – 2000)
15	Flanders	Donkvijver	September	22	9.27	0	
16	Flanders	Donk	May	17	8.6	0	
17	Flanders	Donk	June	22	8.67	0	
18	Flanders	Donk	July	24.5	8.88	0	
19	Flanders	Donk	August	21.2	9.19	0	
20	Flanders	Donk	September	24.2	9.33	0	
21	Flanders	Knokke Heist	May	22.1	8.31	0	
22	Flanders	Knokke Heist	June	21.3	8.22	110	(18 – 410)
23	Flanders	Knokke Heist	July	22.8	8.17	210	(45 – 420)
24	Flanders	Knokke Heist	August	24.2	8.18	>11000	(4200 – 400000)
25	Flanders	Knokke Heist	September	19	8.23	240	(42 – 1000)
26	Wallonia	Butgenbach	May	14.3	7.19	0	
27	Wallonia	Butgenbach	June	21.6	7.87	0	
28	Wallonia	Butgenbach	July	21	8.23	0	
29	Wallonia	Butgenbach	August	17.8	7.22	0	
30	Wallonia	Butgenbach	September	18	7.52	0	
31	Wallonia	Robertville	May	13.1	6.96	0	
32	Wallonia	Robertville	June	22.4	8.68	0	
33	Wallonia	Robertville	July	21.1	8.85	0	
34	Wallonia	Robertville	August	17.9	9.57	0	

N° Sampling	Region	Location	Month	Temperature (°C)	pH	*Vibrio* spp. CFU/100mL (range)
35	Wallonia	Robertville	September	17.5	9.56	0
36	Wallonia	Warfaaz	May	14.2	6.92	0
37	Wallonia	Warfaaz	June	22.9	6.79	0
38	Wallonia	Warfaaz	July	20.7	6.72	0
39	Wallonia	Warfaaz	August	19.3	6.88	0
40	Wallonia	Warfaaz	September	17.4	7.76	0

Table S2 : Values of conductivity in mS/cm and of the fecal indicators *E. coli* and *Enterococci* given by the "Vlaamse Milieumaatschappij Sturing en Rapportering Water Monitoring waterkwaliteit" and the "Service public de Wallonie, department of environment and water" . You have to notice that data for Donk were not available for 2021 so, conductivity and salinity values from 2022 were taken as reference. ND=not determined.

**Water body**	**Date of sampling**	**Température (°C)**	**Conductivity (mS/cm)**	**Salinity (PSU)**	***E.coli* (CFU/100mL)**	***Enterococci* (CFU/100mL)**
**Boerekreek**	2021-09-07	22.9	2.6	1.4	127	125
**Boerekreek**	2021-08-24	21.9	2.5	1.4	94	94
**Boerekreek**	2021-08-10	20 ;6	2.5	1.4	144	94
**Boerekreek**	2021-07-27	21.6	2.6	1.4	177	61
**Boerekreek**	2021-07-13	21.3	2.5	1.4	110	109
**Boerekreek**	2021-06-29	20.7	2.6	1.4	46	77
**Boerekreek**	2021-06-15	24	2.7	1.4	15	61
**Boerekreek**	2021-06-01	22.6	2.5	1.3	46	15
**Boerekreek**	2021-05-17	14.5	2.5	1.6	61	15
**Blaarmeersen**	2021-09-22	19.3	0.4	0.2	0	0
**Blaarmeersen**	2021-09-06	21.7	0.4	0.2	126	15
**Blaarmeersen**	2021-08-30	19.9	0.4	0.2	77	15
**Blaarmeersen**	2021-08-23	20.6	0.4	0.2	127	77
**Blaarmeersen**	2021-08-16	20.1	0.4	0.2	45	15
**Blaarmeersen**	2021-08-09	20.7	0.4	0.2	46	15
**Blaarmeersen**	2021-08-02	20.9	0.4	0.2	30	15
**Blaarmeersen**	2021-07-26	22.9	0.4	0.2	412	46
**Blaarmeersen**	2021-07-20	21.7	0.4	0.2	15	15
**Blaarmeersen**	2021-07-12	21.9	0.4	0.2	30	15

**Blaarmeersen**	2021-07-05	20.7	0.4	0.2	30	15
**Blaarmeersen**	2021-06-28	21	0.4	0.2	46	15
**Blaarmeersen**	2021-06-21	22.8	0.4	0.2	93	15
**Blaarmeersen**	2021-06-14	24.4	0.4	0.2	30	15
**Blaarmeersen**	2021-06-07	21.4	0.4	0.2	15	15
**Blaarmeersen**	2021-05-31	20	0.4	0.2	15	15
**Blaarmeersen**	2021-05-25	15	0.4	0.2	61	15
**Blaarmeersen**	2021-05-17	15.3	0.4	0.2	15	15
**Blaarmeersen**	2021-05-11	16.3	0.4	0.2	251	215
**Blaarmeersen**	2021-05-03	13.5	0.4	0.2	15	15
**Donkvijver**	2021-09-06	21	0.4	0.2	15	15
**Donkvijver**	2021-08-23	20.2	0.4	0.2	15	15
**Donkvijver**	2021-08-09	20.2	0.4	0.2	30	15
**Donkvijver**	2021-07-26	23.2	0.4	0.2	30	30
**Donkvijver**	2021-07-12	21.7	0.4	0.2	46	15
**Donkvijver**	2021-06-28	20.9	0.4	0.2	46	30
**Donkvijver**	2021-06-14	24	0.4	0.2	30	15
**Donkvijver**	2021-05-31	19.6	0.5	0.3	15	15
**Donkvijver**	2021-05-17	15	0.5	0.3	61	110
**Donkvijver**	2021-05-03	13.5	0.5	0.3	15	15
**Knokke**	2021-09-10	ND	ND	ND	27	3
**Knokke**	2021-09-06	ND	ND	ND	5	3
**Knokke**	2021-08-31	ND	ND	ND	3	2
**Knokke**	2021-08-27	ND	ND	ND	16	21
**Knokke**	2021-08-23	ND	ND	ND	88	80
**Knokke**	2021-08-17	ND	ND	ND	14	26
**Knokke**	2021-08-13	ND	ND	ND	61	28
**Knokke**	2021-08-09	ND	ND	ND	12	5
**Knokke**	2021-08-03	ND	ND	ND	30	10
**Knokke**	2021-07-30	ND	ND	ND	86	49

**Knokke**	2021-07-26	ND	ND	ND	49	28
**Knokke**	2021-07-20	ND	ND	ND	0	5
**Knokke**	2021-07-16	ND	ND	ND	27	9
**Knokke**	2021-07-12	ND	ND	ND	11	11
**Knokke**	2021-07-06	ND	ND	ND	35	28
**Knokke**	2021-07-02	ND	ND	ND	18	3
**Knokke**	2021-06-28	ND	ND	ND	11	4
**Knokke**	2021-06-22	ND	ND	ND	10	2
**Knokke**	2021-06-18	ND	ND	ND	1	1
**Knokke**	2021-06-14	ND	ND	ND	16	3
**Knokke**	2021-06-08	ND	ND	ND	10	1
**Knokke**	2021-06-04	ND	ND	ND	5	9
**Knokke**	2021-05-17	ND	ND	ND	1	2
**Donk**	2022-09-22	17.4	0.4	0.2	ND	ND
**Donk**	2022-09-15	20.7	0.4	0.2	ND	ND
**Donk**	2022-09-12	23.4	0.4	0.2	ND	ND
**Donk**	2022-09-05	24	0.4	0.2	ND	ND
**Donk**	2022-08-29	22.3	0.4	0.2	ND	ND
**Donk**	2022-08-22	24.8	0.4	0.2	ND	ND
**Donk**	2022-08-16	25.7	0.4	0.2	ND	ND
**Donk**	2022-08-08	24.6	0.4	0.2	ND	ND
**Donk**	2022-08-04	24.2	0.4	0.2	ND	ND
**Donk**	2022-08-01	23.1	0.4	0.2	ND	ND
**Donk**	2022-07-25	24.4	0.4	0.2	ND	ND
**Donk**	2022-07-18	24.5	0.4	0.2	ND	ND
**Donk**	2022-07-12	24.3	0.4	0.2	ND	ND
**Donk**	2022-07-05	23.2	0.4	0 ;2	ND	ND
**Donk**	2022-06-27	21.3	0.4	0.2	ND	ND
**Donk**	2022-06-20	21.8	0.4	0.2	ND	ND
**Donk**	2022-06-13	21.9	0.4	0 ;2	ND	ND

**Donk**	2022-06-07	19.6	0.4	0.2	ND	ND
**Donk**	2022-05-30	20.6	0.4	0.2	ND	ND
**Donk**	2022-05-23	21.7	0.4	0.2	ND	ND
**Donk**	2022-05-16	20.6	0.4	0.2	ND	ND
**Donk**	2022-05-09	18.7	0.4	0.2	ND	ND
**Donk**	2022-05-02	17.1	0.4	0.2	ND	ND
**Donk**	2022-04-19	15.7	0.4	0.2	ND	ND
**Butgenbach**	2019	25	0 .1	0.05	ND	ND
**Butgenbach**	2021-05-25	ND	ND	ND	15	30
**Butgenbach**	2021-06-07	ND	ND	ND	15	15
**Butgenbach**	2021-06-21	ND	ND	ND	161	109
**Butgenbach**	2021-07-05	ND	ND	ND	15	15
**Butgenbach**	2021-07-22	ND	ND	ND	15	<15
**Butgenbach**	2021-08-02	ND	ND	ND	109	<15
**Butgenbach**	2021-08-16	ND	ND	ND	15	15
**Butgenbach**	2021-08-30	ND	ND	ND	15	<15
**Butgenbach**	2021-09-13	ND	ND	ND	15	<15
**Robertville**	2019	25	0.12	0.06	ND	ND
**Robertville**	2021-05-25	ND	ND	ND	61	15
**Robertville**	2021-06-07	ND	ND	ND	<15	61
**Robertville**	2021-06-21	ND	ND	ND	61	309
**Robertville**	2021-07-05	ND	ND	ND	<15	30
**Robertville**	2021-07-22	ND	ND	ND	<15	61
**Robertville**	2021-08-02	ND	ND	ND	46	61
**Robertville**	2021-08-16	ND	ND	ND	<15	61
**Robertville**	2021-08-30	ND	ND	ND	30	30
**Robertville**	2021-09-13	ND	ND	ND	<15	61
**Warfaaz**	ND	ND	ND	ND	ND	ND
